# Objective Assessment of Porcine Voice Acoustics for Laryngeal Surgical Modeling

**Published:** 2021-05-14

**Authors:** Patrick Schlegel, Kirsten Wong, Mamdouh Aker, Yazeed Alhiyari, Jennifer Long

**Affiliations:** 1 Department of Head & Neck Surgery, David Geffen School of Medicine, University of California Los Angeles (UCLA), Los Angeles, CA 90095, USA; 2 Surgery and Perioperative Careline, Greater Los Angeles VAHS, Los Angeles, CA 90073, USA

**Keywords:** Yucatan mini pigs, parameters, vocal folds, larynx, acoustics, surgery

## Abstract

Pigs have become important animal models in voice research. Several objective parameters exist to characterize the pig voice, but it is not clear which of them are sensitive to the impaired voice quality after laryngeal injury or surgery. In order to conduct meaningful voice research in pigs, it is critical to have standard functional voice outcome measures that can distinguish between normal and impaired voices. For this reason, we investigated 17 acoustic parameters before and early after surgery in three Yucatan mini pigs. Four parameters showed consistent changes between pre- and post-surgery recordings, mostly related to decreased spectral energy in higher frequencies after surgery. We recommend two of these, 50% spectral energy quartile (Q50) and Flux, for objective functional voice assessment of pigs undergoing laryngeal surgery. The long-term goal of this process is to enable quantitative voice outcome tracking of laryngeal surgical interventions in porcine models.

## Featured Application

Matlab and Praat scripts for parameter calculation are part of the supporting information and may be used to replicate all parameters discussed in this work.

## 1. Introduction

Voice impairment is a major factor in public health, affecting the economic prosperity of society as a whole, as well as the social functioning and quality of life of individuals [1–3]. Roy et al. interviewed a random sample of 1326 adults in Iowa and Utah and found that the lifetime prevalence of a voice disorder was 29.9%. Of the interviewed participants, 7.2% reported an absence from work for one day or more due to their voice during the last year [1]. Another study by Cohen et al., reviewing data of 386 patients with a short-term disability claim due to a laryngeal disorder, found that voice disorders were associated with an average missed work attendance of 39.2 days in 12 months. This resulted in an average wage loss of $4437.89 per patient [2]. The effect on quality of life was shown by Marmor et al. One of their findings was that patients with reported voice problems experienced nearly twice the likelihood of depressive symptoms [3].

Accordingly, the human voice is an ongoing subject of research with novel treatments under development for many human voice disorders [4–7]. Testing new treatments obviously carries certain risks, and translating research into humans often requires pre-clinical animal models to demonstrate safety and efficacy before enrolling human subjects. Due to their similar anatomy, physiology, and genetics, pigs have emerged as an important animal model. Additionally, they are easy to breed, have no complex husbandry needs, and produce large litters. In biomedical research, miniature pigs are particularly useful because their full adult size is more manageable than traditional farm pigs [8,9]. For voice research, pig and human vocal folds exhibit similar physical characteristics, most notably the vocal fold thickness and distribution of elastic and collagen fibers. Pigs are also highly vocal in a similar frequency range as humans, unlike other animal models such as rabbits and small rodents [10–12].

However, understanding the voice in non-humans has complexities, as there are obvious differences between pig and human vocalizations. Besides the anatomic differences of tongue position, position of the larynx, and vocal tract length, pig vocalizations exhibit nonlinear qualities such as subharmonics and chaotic episodes that occur without complex nervous system control [13,14]. Pigs also cannot be instructed to phonate on command, to hold a vowel, or to phonate using a variety of call types. In general, lower frequency calls like grunts are highly variable and may be attributed to non-laryngeal origins [11], although there is no unanimity on this subject [13]. In contrast to grunts, which are emitted nasally, higher frequency calls like screams and squeals are emitted orally [15] and are in general assumed to originate from the vocal folds [13].

Prior studies have presented various methods of classifying normal porcine phonation, differentiating between call types in an agricultural and animal communication context. However, no unanimous differentiation rules have emerged [11,15–18]. Considerable blurring between call types is evident due to the indiscrete nature of the calls and their existence on a sound continuum [16,18]. Extensive variation in “normal” pig calls is also widely documented [11,15]. Other complexities include noisy irregular data, pigs moving during phonation, nonlinear distortion, and recording noise, which complicate interpretation [14]. Further, results of these studies assessing more natural pig phonation are not necessarily transferable to the situation of pigs undergoing laryngeal surgery, i.e., in a post-surgery setting. For these reasons, we avoid a more specific differentiation between call types in this work and focus on higher frequency calls such as squeals and screams in general.

Few studies have addressed porcine voice function after laryngeal tissue engineering. In the context of vocal fold surgery on pigs, two notable studies need to be mentioned: Ansari et al., implanted hemi-larynx replacements into six White/Landrace cross-bred pigs [7]. Squeal and grunt voice samples were collected but spectrograms remained altered six months after surgery. Brookes et al., implanted a different hemi-larynx replacement in three Yucatan mini-pigs [19]. They reported phonation amplitude of isolated squeals measured at a fixed microphone distance and found recovery of baseline amplitude after eight weeks. However, other voice quality measures were not assessed. These studies illustrate the need for further research in quantitative porcine acoustic analysis relevant to laryngeal surgery.

Various software tools exist to examine human phonation and voice, but tools designed for human voices do not necessarily work on pigs. Common acoustic parameters including Jitter, Shimmer, and many noise measures are intended for analysis of sustained phonation with distinct fundamental frequency (F0) [20,21]. Porcine phonation, in contrast, is often aperiodic, so these parameters are not expected to generate reliable or meaningful results [15–17]. Furthermore, although voice parameters provide the possibility of objective voice assessment, many of them fail to reliably correlate with perceptual voice quality judgments [22]. Jitter and Shimmer in particular have been questioned due to the fact that they are poorly distinguished by auditory perception [23]. Objective parameters still serve a purpose in providing objectivity and uncovering subtle properties of acoustic signals, but parameters suitable for aperiodic voices are needed to evaluate pigs.

Some parameters have been previously described for aperiodic pig voices and were used to differentiate between pig phonation types [15–17]. Tallet et al., in 2013 analyzed the piglet vocalization repertoire of 1513 calls from 84 (Large White × Landrace) × (Duroc × Pietrain) piglets in different behavioral contexts. They identified a set of eight parameters using a cluster analysis approach that differentiated best between two or five types of calls [16]. Garcia et al., analyzed calls from 19 wild boars. Out of 19 acoustic parameters calculated using PRAAT and the MIR Toolbox in MATLAB, four parameters were selected to best differentiate between four call types [17]. In contrast, Linhart et al. investigated parameter change with emotional state in 88 Large White × Landrace piglets. They used four parameters calculated with Avisoft SASLab and PRAAT and found distinct correlations with emotion in squeals and grunts [15]. Further, all of these authors mention the problem of not clearly determinable pig phonation F0 from acoustics and only used parameters suited for aperiodic voice [15–17].

All of these parameters were previously only used to differentiate between call types of healthy pigs (or boars). To the best of our knowledge, no parameters have been introduced that were explicitly designed or tested to differentiate between pig voice before and after laryngeal injury or surgery. We have observed by acoustic perception that early after vocal fold surgery, pig vocalizations become more “grunt-like”. We therefore hypothesize that the parameters identified in these three previous works may also be able to differentiate between pre- and post-surgery squeals. For this reason, we implemented seven parameters that were used by Tallet et al. [16], three parameters by Garcia et al. [17], and three additional parameters used by Linhart et al. [15] as well as the length of the call, which was included in all three studies [15–17]. For demonstrative purposes, we also calculated three classical F0-based human voice measures (Jitter (%) (Jit), Shimmer (%) (Shim), and Cepstral peak prominence (CPP)). Since no exact calculation algorithms were given in these previous works, it cannot be guaranteed that the parameters are perfectly reproduced. We aim to improve this reproducibility deficit by providing our computer code as Supplemental Materials.

Therefore, the aims of this work can be summarized as follows:

1. Identify acoustic parameters that reflect voice changes after vocal fold implantation surgery in pigs.
2. Investigate the changes in parameters and associated changes in acoustic signals between pre- and post-surgery.
3. Enhance voice research reproducibility by making all code freely available, allowing for exact replication of our parameter implementations.

These goals are met by analysis of pre- and post-surgery pig squeals using 14 parameters for aperiodic pig voice and three classical F0-based voice measures. A detailed discussion on statistically significant changes and trends is given. This work demonstrates important progress in our long-term goal of developing a reliable pre-clinical animal model for evaluating voice function after laryngeal surgery.

## 2. Materials and Methods

UCLA’s Institutional Animal Care and Use Committee approved this work, performed in accordance with AALAC and USDA guidelines. Three Yucatan mini-pigs (two male, one female) underwent laryngeal cordectomy and vocal fold replacement surgery as described below. Acoustic recordings of spontaneous vocalizations were collected before and after surgery; segments of high-pitched phonation were selected for analysis. We did not differentiate between squeals and screams since after surgery both are not clearly distinguishable, and differentiation between these call types in literature is also very blurred [11,15–18]. For simplification in the following, we refer to all selected squeals, screams, and high-pitched phonation segments as “squeals”.

As summarized in Table 1, the age range of the pigs was 12–34 weeks, and the weight range was 13–35 kg. For each pig, at least 31 normal squeals were recorded before vocal fold implantation surgery (see Section 2.1. Surgical Procedure). At least 42 abnormal squeals were collected within one week after surgery. Only squeals classified as low noise were chosen for analysis (see Section 2.2. Acoustic Recordings).

### 2.1. Surgical Procedure

Each Yucatan mini pig was sedated with intramuscular Telazol (tiletamine) to enable intravenous catheter placement in the tail vein. Anesthesia was then induced with inhaled isoflurane and intravenous propofol. A size 6–0 endotracheal tube was placed by an experienced veterinary technician. A vertical midline neck incision was made to expose the laryngeal cartilage. The endolarynx was accessed by incising the thyrohyoid membrane for a superior pharyngotomy approach. This enabled direct visualization of both vocal folds, with the endotracheal tube positioned posteriorly. The membranous cover layer was then resected from one or both true vocal folds by sharp dissection. Resection extended from just anterior to the arytenoid cartilage, to just posterior to the anterior commissure, leaving 1–2 mm of normal vocal fold at each border. Mucosa was removed down to the thyroarytenoid muscle, equivalent to a European Laryngological Society type 2 cordectomy. Vocal fold implants containing adipose-derived stem cells in a fibrin scaffold were secured onto the defect. The implant details have been previously described and are not the focus of this paper [24]. Implants were secured with anterior and posterior sutures of 5–0 plain gut. All animals tolerated surgery well, returned to ambulatory state within minutes after emerging from anesthesia, and proceeded to oral diet the same day.

### 2.2. Acoustic Recordings

**Figure 1.**
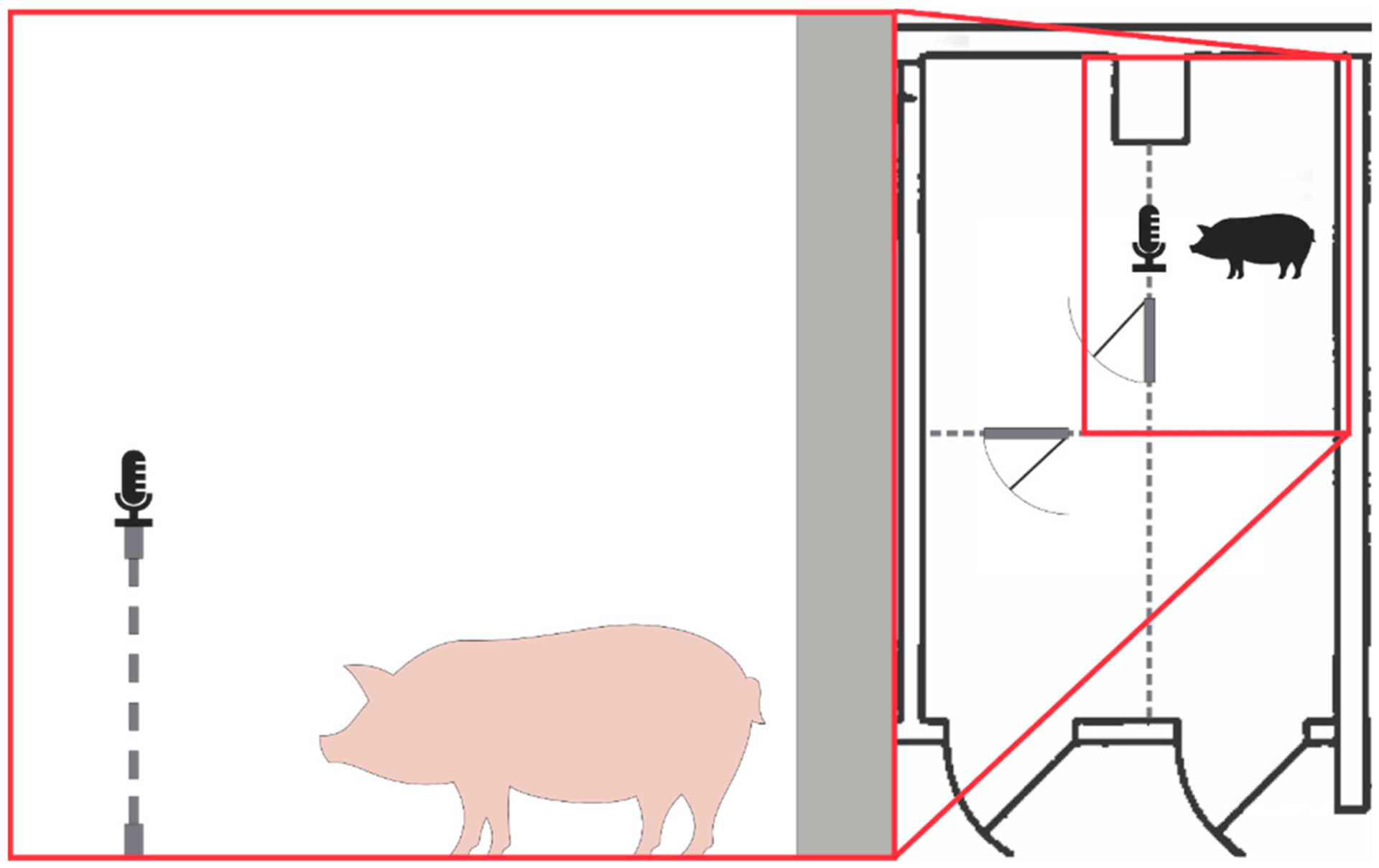
Schematic illustration of the pig recording environment.

Recording of squeals was performed using an H4n Pro digital audio recorder (140 dB SPL, minimum sensitivity −12dB, 16 bit, 44,100 Hz sampling rate, stereo). As illustrated in Figure 1, the digital audio recorder was secured at the entrance of the pig enclosure (7.43 m^2^ area) about 0.75 m above the ground to record the pig’s natural phonations. During pre- and post-surgery recording sessions, one pig at a time was housed for a 6 to 48 h period in the vivarium housing, and all vocalizations were recorded. The pigs were free to roam, eat, and sleep during this time.

From the recorded audio material, 95 normal squeals (collected before surgery) and 143 abnormal squeals (collected after surgery) were manually extracted. Vocalizations were identified as squeals based on the following criteria: duration of sound at least 0.3 s, no overlapping vocalizations, and high subjective pitch. Starting and ending positions of each squeal were determined by plotting the relevant acoustic signal sections in Matlab (version R2020b). Afterwards, all extracted squeals were rated on a scale of 0 to 2 (low to high background noise) by three raters in one rating session. All squeals with an average rating above 1.00 were excluded from analysis. Additionally, squeals were investigated for not clearly audible artifacts (such as slight overdrive), and distorted squeals were discarded. This resulted in 67 “normal” squeals, recorded before surgery, and 79 “abnormal” squeals recorded on up to two different dates after surgery (see Table 1).

In Data S1, one example squeal for each pig pre- and post-surgery is given (six squeals total).

### 2.3. Parameter Analysis

For parameter calculation, recordings were converted to mono by discarding the right audio channel and scaled to −1, 1 range as no absolute volume could be determined in our recording environment. In total, 14 different parameters and versions of parameters that were previously used on aperiodic pig voice were chosen. Additionally, we included three classic F0-based voice measures. We mainly implemented parameters that were previously described to differentiate squeals from other pig phonation, as squeals generally seemed to become more “grunt-like” (but still distinguishable from grunts) after surgery [15–17].

In total, 13 parameters were implemented in Matlab (version R2020b). Harmonics-To-Noise ratio was calculated using Praat (version 6.1.38), as described in [15]. Jit, Shim, and CPP were also calculated using Praat with default settings with exception of the “voicing threshold”-setting, which was set to 0 to allow for F0 detection in the entire squeal (otherwise calculation of these parameters would not have been possible for some squeals). While great care has been taken to implement the parameters exactly as described in their respective sources, exact reproduction cannot be guaranteed, in part due to incomplete descriptions in the source material. To enhance the reproducibility of our work, the Praat script and Matlab code used to calculate these parameters in this work are provided in the supporting information (Files S1–S3). Names, abbreviations, sources, units, and a brief description of all parameters are listed in Table 2. Table S1 tabulates the full dataset of parameter values for all squeals and pigs.

**Figure 2.**
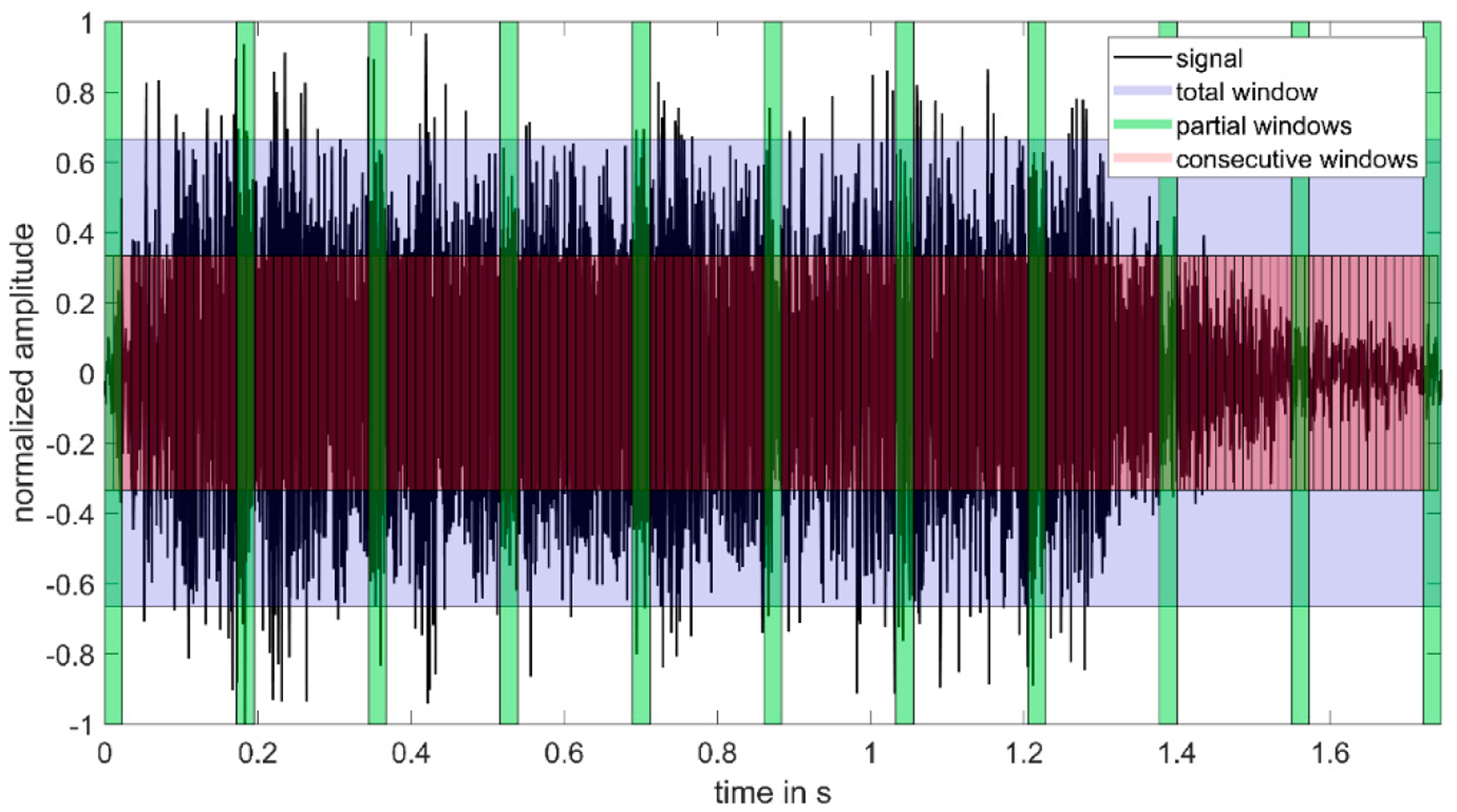
Illustration of different types of windows. “Total” refers to a single window including the entire signal, “partial” refers to single windows only containing small subsections of the signal, and “consecutive” refers to multiple consecutive, overlapping windows.

Some of the parameters are similar and differ mainly in the windowing of the signal or the signal sections on which they are calculated. In Figure 2, we illustrate the varying types of time windowing (total, partial, and consecutive) for one example squeal. “Total” refers to a parameter calculated on one single window including the entire squeal, “partial” refers to a parameter only calculated for a small subsection of the signal in time, and “consecutive” refers to a parameter calculated for multiple consecutive, overlapping windows that is then averaged to obtain the final parameter value for the squeal.

### 2.4. Statistical Analysis

Parameter values were calculated for each squeal, and statistical analysis was performed in Matlab. The parameters Jit, Shim, and CPP were excluded from statistical analysis as we do not expect that they notably change between pre- and post-surgery. Including these parameters in the statistical analysis would have led to a lower overall statistical power due to the required correction for multiple comparisons. For this reason, only mean values and standard deviations were calculated for these three parameters for demonstration purposes.

For comparison of parameter median values of pre- and post-surgery squeals, Wilcoxon rank sum tests were chosen. We wanted to avoid two-sided tests since in such scenarios, a statistically significant difference in medians is always found as long as the number of samples is sufficiently large [26]. Further, the power of the test would have been unnecessarily decreased for the two-sided case. For this reason, we formulated a one-sided h0 hypothesis for each parameter that reflected whether we expected the median to either increase or decrease after surgery. All hypotheses were formulated using only raw data of squeal acoustic signals, spectrums and spectrograms before performing any statistics. These hypotheses and the reasoning behind them can be found in Table S2.

Tests were performed separately for all three pigs comparing the pre-surgery squeals with post-surgery recordings. Animals were not compared with each other since the main objective of this work is to find objective parameters that differ between normal and injured state for each animal. Further, animals had different ages and sexes, as well as inherently different vocal features such that their acoustic “vocoprints” differed at baseline.

**Figure 3.**
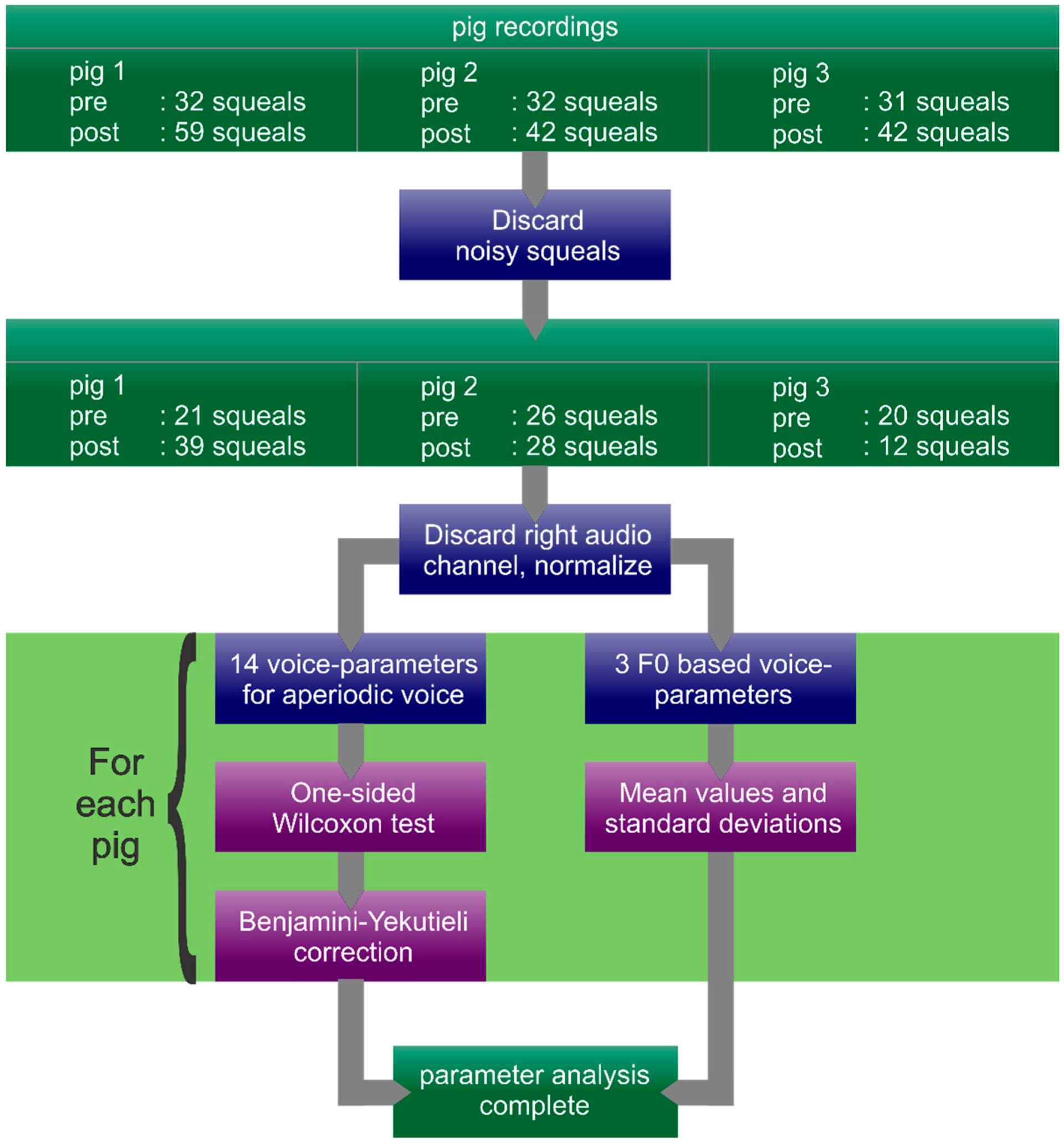
Step-by-step illustration of the data analysis process.

For the calculated p-values, we controlled the false discovery rate at 5% for each pig (i.e., the expected percentage of false positive tests). For this we chose the Benjamini–Yekutieli procedure [27], as we have to assume that there may be unknown dependencies between the different parameters. A step-by-step illustration of the data analysis process is depicted in Figure 3.

## 3. Results

In total, 146 squeals from the three pigs did demonstrated variable features among the subjects. Table 3 summarizes the statistical analysis, with abbreviations of all parameters with statistically significant changes between before and after surgery. No parameters exhibited statistically significant change in all three pigs. Q50 and Flux changed in the two male pigs but did not reach statistical significance in the female. Q50_min_ changed statistically significantly in the female and one male pig. Parameters changed most prominently in pig 3, which was also the only pig with highly significant changes (*p* ≤ 0.001). Corrected *p*-values for all parameters and comparisons can be found in Table S3 in the supporting information.

Table 4 shows mean values and standard deviations for F0-based voice measures. These parameters seem to vary randomly with no clear changes in any direction. Jit exhibits the most notable differences on average between pre- and post-treatment, but not uniformly for all pigs.

**Figure 4.**
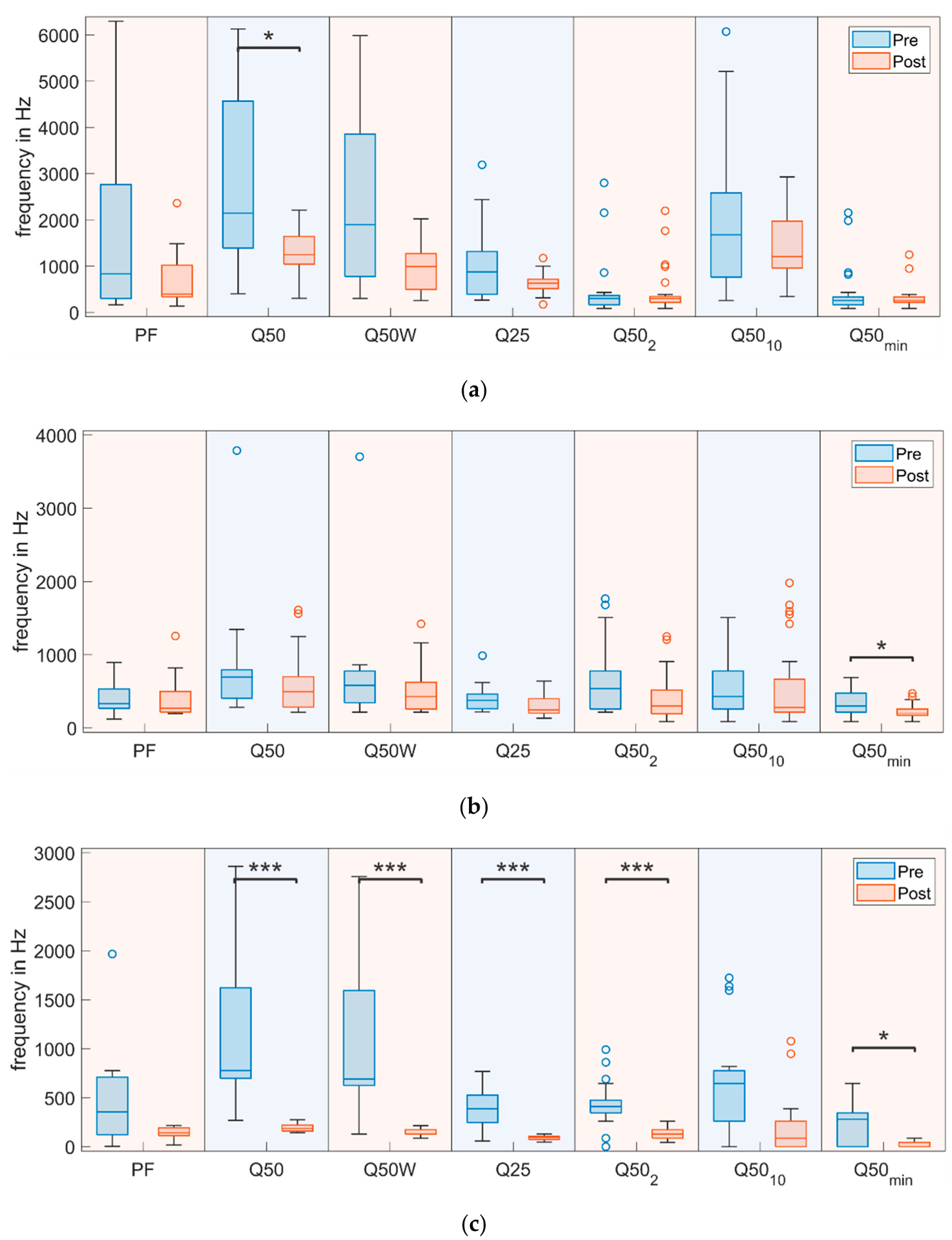
Boxplots of frequency-based parameters for (**a**) pig 1, (**b**) pig 2, and (**c**) pig 3 for pre- and post-surgery recordings. Statistically significant changes are marked with * symbols. (*p ≤ 0.05, *** *p* ≤ 0.001).

In Figure 4, boxplots for all frequency-based parameters (from PF to Q25, see Table 2) are given for all three pigs (from (a) pig 1 to (c) pig 3). The “box” of each boxplot denotes the inter quartile range (from the first to the third quartile) with the horizontal line indicating the position of the median. The whiskers are calculated according to Matlab default settings as a multiple of the inter quartile range extending to the farthest data point within the potential whisker range. Everything above or below the whiskers is marked as an outlier.

Statistically significant changes are marked with * symbols analogously to Table 3. In general, median values for all these parameters decreased after surgery However, those parameters based on eleven partial windows (Q50_2_, Q50_10_ and Q50_min_) changed less consistently. Overall, these findings reflect a condensed frequency range after surgery in all pigs. Pig 2 (the only female) exhibited the smallest initial frequency range and also the smallest change after surgery.

**Figure 5.**
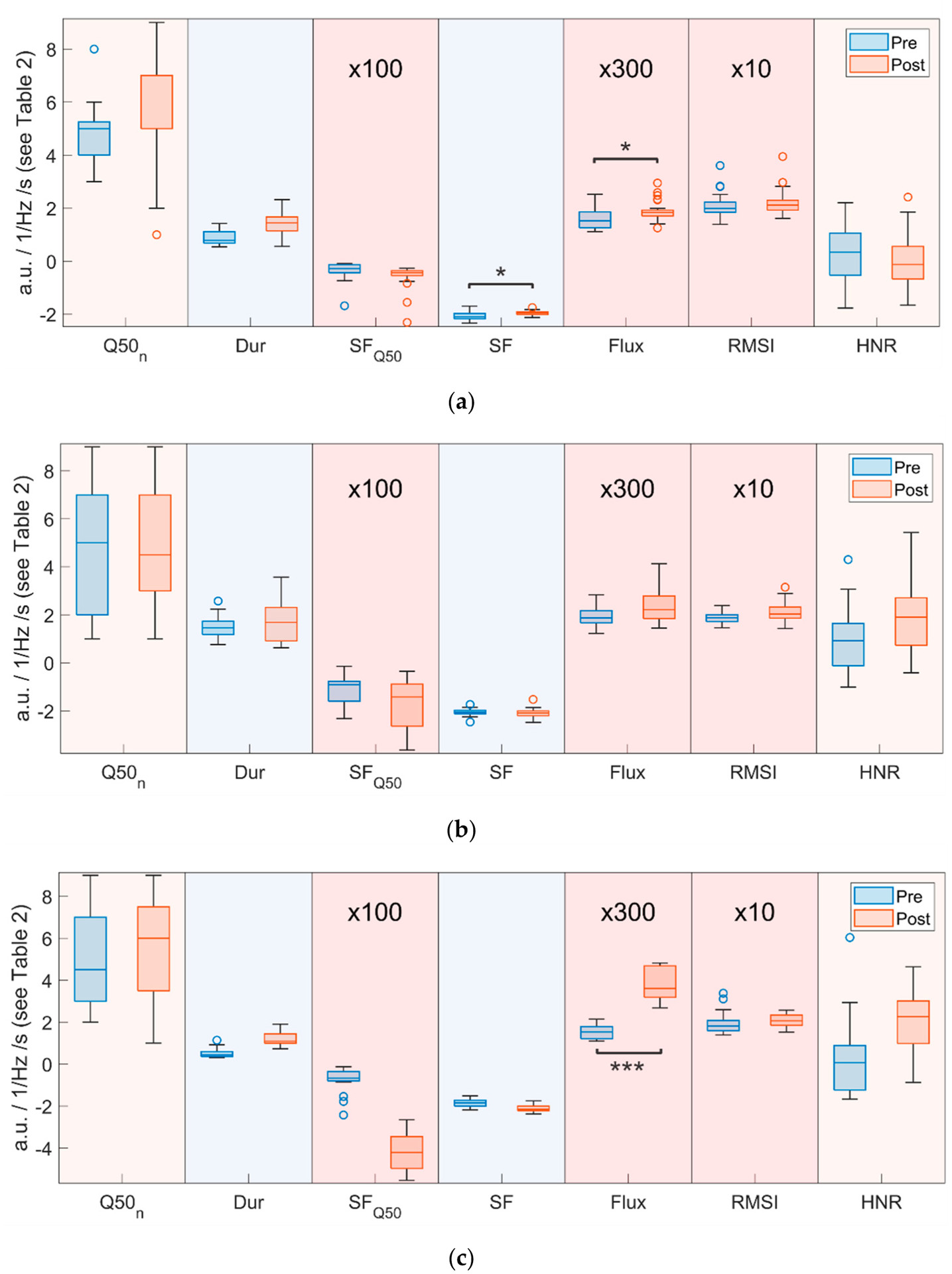
Boxplots of remaining parameters for (**a**) pig 1, (**b**)pig 2, and (**c**) pig 3 for pre- and post-surgery recordings. SF_Q50_ is scaled by a factor of 100, Flux by a factor of 300, and RMSI by 10. Statistically significant changes are marked with * symbols. (* *p* ≤ 0.05, *** *p* ≤ 0.001).

Analogously, Figure 5 shows boxplots of the remaining parameters (Q50_n_ to HNR). Values for SF_Q50_, Flux and RMSI were scaled by factors of 100, 300, and 10 to allow depiction of all these parameters in one plot. For the respective parameter units, refer to Table 2. Parameters in this figure change less consistently between pre- and post-surgery; only Flux displays statistically significant change, in more than one pig. Further, contrary to the stated H0 in Table S2, HNR increased for two pigs after surgery and minimally decreased in one.

## 4. Discussion

A voicing animal model is essential to test the safety and efficacy of certain laryngeal interventions for voice disorders before translating to humans. Traditionally, structural and compositional measures such as histology and protein content have dominated the post-operative analysis of surgical methods. This is due in part to the lack of objective methods for assessing non-human voicing. We aim in this work to develop a robust and quantitative voice analysis method to enable functional outcome assessment after porcine laryngeal surgery. Establishing long-term normal voicing in a pig model is an important efficacy benchmark for elective surgeries designed to restore normal voice function in humans.

In this work, pig voices were compared in the normal state and shortly after a disruptive laryngeal surgery (vocal cordectomy and replacement with a tissue-engineered implant.) Voices are perceptually abnormal at the early phase of wound healing, consistent with expectations after such an invasive procedure. The long-term goal of the surgical procedure is recovery of normal voice during the process of wound healing, which is expected to take several weeks or months. Long-term analysis is ongoing and is beyond the scope of this paper. For this work, we focus on distinguishing the acoustic features which are altered early after surgery when the vocal fold microstructure remains notably abnormal.

In the three animals studied here, we developed methods for rapid collection and analysis of spontaneous vocalizations. The resulting dataset of 146 squeals provides ample material for calculating the 17 acoustic parameters studied from each squeal. With this large number of data points, a high degree of variability was observed both within individual pigs and when comparing results across animals. Notably, the intra-subject variability appeared greatest in the pre-operative normal state, indicating a wide range of normal vocal behaviors. Post-operatively, the range of many parameters decreased, suggesting a more limited vocal repertoire in the early post-surgical period. While statistical analysis did not show consistent significant changes in all three pigs, several interesting points were observed that are discussed in the following sections.

### 4.1. F0-Based Parameters

Parameters developed for human voice and based on fundamental frequency (F0) did not show consistent trends for the different pigs between pre- and post-surgery. This is not surprising, as these parameters all depend on a correctly detected F0. Jitter measures the perturbation in cycle lengths, which are calculated based on detected F0 [21]. Shimmer measures amplitude perturbation between consecutive cycles, which also relies on correctly detected cycles, i.e., F0 [21]. CPP measures the prominence of the fundamental quefrency peak in the cepstrum. The fundamental quefrency and thereby the position of this peak are directly related to F0 [25].

Praat and other voice analysis tools may be able to detect an F0 for almost any type of signal if the settings are chosen liberal enough. However, if the signal itself has a certain degree of aperiodicity, as is the case with most pig phonation, this will only result in a mostly random F0 and therefore no meaningful F0-based parameters. Small pig-specific changes as observed here, e.g., Pig 2 Jit decreasing slightly or Pig 3 Jit increasing slightly, are most likely related to subtle patterns within the signals that may influence the F0 detection algorithm but much less to the parameter itself. We therefore strongly advise against the use of F0-based parameters on aperiodic pig voice.

### 4.2. Frequency-Based Parameters

**Figure 6.**
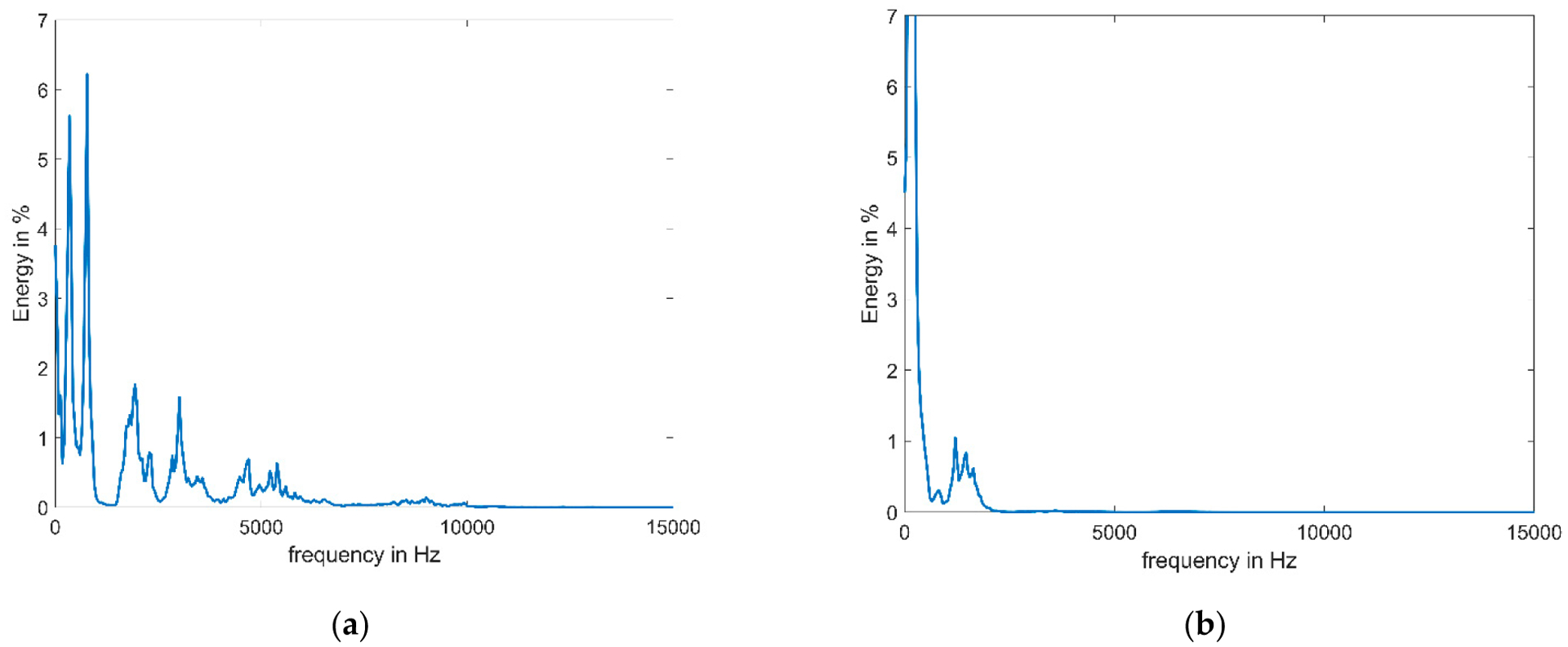
Averaged spectral energy of 10 squeals of pig 3 (**a**) before and (**b**) after surgery. Energy spectra were calculated using 1024 data point windows with 50% overlap. A distinct energy reduction at higher frequencies can be seen, resulting in lower PF and Q-parameters.

Healthy pig squeals often include stronger higher frequencies as shown exemplarily in Figure 6 for the averaged energy spectrum of ten pre- and ten post-surgery squeals for pig 3. This explains the on average lower values of the frequency-based parameters (see Figure 4). PF is the position of the highest peak, which is more often within the lower frequencies since energy within higher frequencies is reduced. Similarly, Q-parameters describe the position at which half (for Q25 25%) of the total energy (in the entire squeal or one window, depending on the parameter) is reached (for details see Table 2). Less of the energy is within the higher frequencies; therefore, these positions are reached earlier, and Q parameters decrease after surgery according to our expectations (see hypotheses in Table S2).

This trend is less consistent in the partial window-based parameters (Q50_2_, Q50_10_, and Q50_min_), since they are only calculated for a fraction of the entire squeal, which results in a lower frequency resolution. Therefore, a more subtle shift of spectral energy may not be shown within these parameters. Further small variations of selected starting and ending-positions between squeals may lead to these parameters being calculated for slightly different areas of each squeal. Furthermore, changes over the duration of the squeal are not shown within partial window-based parameter values; still, Q50_min_ showed a statistically significant change in two pigs, which may hint at some usefulness of these windowed parameters for large datasets with carefully defined start and end-positions of squeals (best using a repeatable, objective method for squeal extraction).

With the exception of PF, all investigated frequency-based parameters are defined very similarly with the general idea of making a simplified similarly statement about spectral energy distribution. For our data, Q50 showed the most consistent change between pre- and post-surgery squeals, which may be due to this parameter being the only one of its type calculated for the entire squeal at once, allowing for maximal frequency resolution. Q50W is similar but somewhat less robust due to windowing and offers no additional insight over Q50 alone. PF on the other hand was less consistent, displaying no statistically significant change, mainly since it describes the position of the absolute maximum of the energy spectrum, which may more easily change. To avoid redundancy, among frequency-based parameters investigated, we found Q50 to be the best choice for objective assessment of pig voice health.

### 4.3. Other Parameters

Most of the remaining parameters did not show a significant change between pre- and post-surgery as can be seen in Figure 5. Q50_n_ can be interpreted as stating the number of the partial window in the signal where higher harmonics are least dominant (excluding edge windows). All values are therefore integers from 2 to 10. Only pig 2 exhibited a subjective change. Similar to the partial window properties discussed above, the reduced data richness of this parameter limits its usefulness. The squeal duration (Dur) also did not show a significant change for all pigs between pre- and post-surgery, contrary to our expectations (see hypotheses in Table S2), even slightly increasing after surgery for all pigs. This finding suggests that squeal duration may be controlled more by behaviors inherent to pig communication rather than by the physiology of the vocal fold state. The slight increase in squeal duration may be meaningful, but more data are needed for verification.

Spectral flatness parameters SF_Q50_ and SF were calculated similarly; the main difference was that SF_Q50_ was scaled using Q50. We expected spectral flatness to increase as post-surgery squeals appear to become “noisier”, but no such trend could be observed in all pigs. The decrease in SF_Q50_ can be largely attributed to the Q50 scaling and therefore has no additional information value.

**Figure 7.**
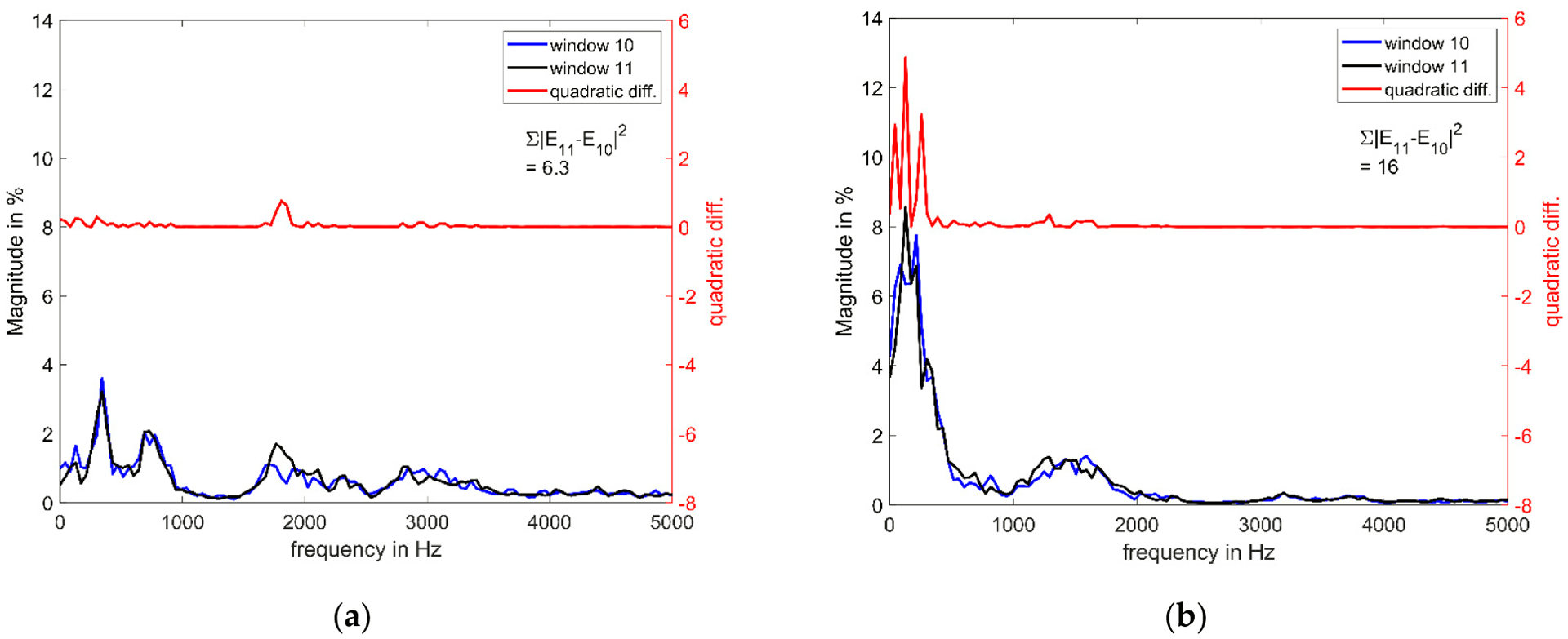
Averaged magnitude of 10 squeals of pig 3 (**a**) before and (**b**) after surgery for two neighboring consecutive windows. The red line is the quadratic difference between both windows at each x-axis position. Magnitude spectra were calculated using 1024 data point windows with 50% overlap. Flux is higher after surgery, as it measures the quadratic difference between % magnitude; i.e., a single high deviation results in a larger total deviation than multiple small ones.

Flux did show a consistent trend of increasing after surgery in all pigs. Acoustically, Flux can be thought of as a time-instability of the voice. Higher values typically indicate greater variation in energy spectra over time. In Figure 7, the averaged normalized magnitude spectrum of 10 squeals pre- and post-surgery is depicted for two neighboring consecutive time windows within the squeal (consecutive windows 10 and 11 with 50% overlap). For the calculation of Flux, first the quadratic difference between these neighboring windows, the red line, is calculated. As can be seen in the figure, this difference is especially large for the steep inclines in the low-frequency range of post-surgery spectra. Furthermore, since the difference is calculated quadratically, a single large deviation between neighboring windows is often bigger than multiple smaller ones. The reduced high-frequency energy that led to decreased Q-parameters also therefore impacts Flux. Flux is the sum of all these differences over frequencies and time and therefore increases after surgery.

We expected RMSI to increase after surgery, as squeals lose structure, leading to less dominant global peaks in the acoustic signal and hence a higher RMSI (see Table S2). RMSI increased for all pigs after surgery on average, but none of these changes was significant.

Interestingly, contrary to our expectations (see Table S2), HNR increased after surgery, indicating, by definition of the parameter, stronger harmonics. However, this is obviously not the case as can be seen in Figure 5, since even in pig 3 with diminishing higher frequencies after surgery, HNR increased. The algorithm of HNR is rather complex and even if it can theoretically be calculated on aperiodic signals, it was not designed for this purpose [15]. The reason for the increase in HNR after surgery may therefore be the following: Even in the most aperiodic of signals, the HNR algorithm still searches for the following: Even in the most aperiodic of signals, the HNR algorithm still searches for the best possible F0 candidate [28]. As the squeals post-surgery lose most of their higher frequencies, this peak may be more consistently found within the same low frequency range for all windows. This leads, in comparison to pre-surgery, to a more dominant “F0” peak and, as the F0 peak is part of the harmonics, to a higher harmonics-to-noise ratio (higher HNR).

To summarize, among these other parameters, only Flux showed consistent behavior between pre- and post-surgery. Dur, RMSI, and HNR showed weaker tendencies and have theoretical limitations.

## 5. Limitations

At this phase of the work, only a small number of pigs were investigated, which the statistical power and generalizability of our results. Confirming our findings with a larger number of animals will be useful. Interestingly, we did note subjectively different vocal behavior in the one female pig studied (pig 2). Her pre-operative vocal frequency distribution was more condensed than the two male pigs; her voice then did not change as dramatically after surgery. It is not yet clear if this represents natural inter-subject variability, if these findings indicate a sex difference in vocal features between male and female pigs, or if the difference is mere coincidence. However, it does illustrate the importance of including both sexes in pre-clinical animal models.

Regarding acoustic data processing, squeals were manually selected for this work. Reproducibility could be affected, as different people may have different preferences for what is and is not a squeal. Further selection of start and endpoints of squeals was done manually, which may have contributed to changes in squeal duration and squeal section-based parameters. We tried to counteract this by applying standards for squeal selection and by rating selected squeals based on noise, also excluding phonation that was retrospectively not considered a squeal by the majority of raters.

More parameters than the ones implemented and investigated in this paper exist [15–17,29]. Reproducibility, especially of more complex parameters, is limited and hence we cannot guarantee that parameters we implemented based on previous work were exactly replicated [30,31]. To ensure an as-close-as-possible replication, we followed each step of the parameter calculation exactly as stated in their respective sources. Further, to allow an easier and more exact replication of our work, we attached all code for parameter calculation in the supporting information (Files S1–S3).

## 6. Conclusions

This study explored the use of parameters that differentiate phonation types in pigs to compare squeals before and after laryngeal surgery. Three classical F0-based voice measures and 14 acoustic parameters for aperiodic pig voice from previous work were investigated. No statistically significant changes for all three pigs could be identified; nevertheless, the parameters Q50 and Flux show consistent and acoustically-relevant trends between pre- and post-surgery squeals. The parameters RMSI and HNR show weaker tendencies. The observed decrease of high frequency energy early in the post-operative period is reflected in decreased Q50 and increased Flux and HNR. The degradation of over-all squeal structure is partially reflected in increased RMSI. Thereby, objective assessment of porcine vocal function after surgery and recovery may be possible using these parameters. Algorithms and scripts for the calculation of all parameters are provided in the supporting information to ensure accessibility to researchers and reproducibility.

## Supplementary Material

Supplementary material

## Figures and Tables

**Table 1.** Sex, age, weight, and number of recorded and analyzed squeals for all pre- and post-surgery recording sessions for each pig.

Pig	Sex	Age	Weight	Normal Squeals	Abnormal Squeals
1	M	12 weeks	13 kg	32 (21)^1^	59 (39)
2	F	19 weeks	25 kg	32 (26)	42 (28)
3	M	34 weeks	35 kg	31 (20)	42 (12)

1 number of recorded squeals and number of selected squeals (in parentheses).

**Table 2.** Name, reference, units, general type of windowing, and a brief description of each investigated parameter.

Parameter and Reference	Abbreviation	Unit	Windowing	Description
Peak frequency [16]	PF	Hz	total	Position of the highest peak in the energy spectrum of the entire signal.
50% energy spectrum quantile [16]	Q50	Hz	total	Frequency that divides the energy spectrum of the entire signal in two intervals of equal energy.
50% first window quantile [16]	Q50_2_	Hz	partial	Eleven evenly spaced hamming windows are calculated within the signal. Q50_2_ is the Q50 of the second window.
50% last window quantile [16]	Q50_10_	Hz	partial	Eleven evenly spaced hamming windows are calculated within the signal. Q50_10_ is the Q50 of the second to last window.
Minimum 50% window quantile [16]	Q50_min_	Hz	partial	Eleven evenly spaced hamming windows are calculated within the signal. Q50_min_ is the lowest Q50 of the nine inner windows.
50% energy spectrum quantile [15]	Q50W	Hz	partial	Frequency that divides the average energy spectrum of all windows of the entire signal in two intervals of equal energy.
25% energy spectrum quantile [17]	Q25	Hz	partial	Frequency that divides the energy spectrum of the entire signal in two intervals of 25% and 75% energy. Calculated for multiple spectral energy windows and averaged.
Duration [15–17]	Dur	s	total	Duration of the entire squeal.
Maximum 50% window quantile position [16]	Q50_n_	a.u.	partial	Eleven evenly spaced hamming windows are calculated within the signal. Q50_n_ is the number of the window with the highest Q50 of the nine inner windows.
Spectral Flatness [17]	SF	a.u.	consecutive	Describes how close the spectrum is to the spectrum of white noise. Calculated for multiple spectral energy windows and averaged.
Spectral Flatness Q50 [16]	SF_Q50_	1/Hz	consecutive	Spectral Flatness calculated for the energy spectrum of the entire signal and divided by Q50.
Spectral Flux [17]	Flux	a.u.	consecutive	Average difference in energy between neighboring energy spectrum windows.
Root-Mean-Square sound intensity [15]	RMSI	a.u.	total	Root mean square of normalized signal (since no accurate sound pressure could be measured).
Harmonics-to-Noise ratio [15]	HNR	d.B.	total	Represents the degree of acoustic periodicity.
Jitter (%) [21]	Jit	a.u.	total	Measures period perturbation
Shimmer (%) [21]	Shim	a.u.	total	Measures amplitude perturbation
Cepstral peak prominence [25]	CPP	d.B.	total	Prominence of the quefrency peak in the cepstrum, measures noise

**Table 3.** Statistically significantly changing parameters for all pre/post-surgery comparisons.

Pig#	Pre/Post *p* ≤ 0.05
1 (M)	Q50*,SF*,Flux*
2 (F)	Q50_min_*
3 (M)	Q50***, Q50_2_***, Q50_min_*, Q25***, Flux***, Q50W***

* *p* ≤ 0.05;

*** *p* ≤ 0.001.

**Table 4.** Mean and standard deviation (std) of F0-based parameters for all pigs pre- and post-surgery.

Pig#	Jit [Mean]	Jit [Std]	Shim [Mean]	Shim [Std]	CPP [Mean]	CPP [Std]
1 pre	0.083	0.013	0.205	0.017	14.218	1.396
1 post	0.091	0.011	0.206	0.018	15.181	2.000
2 pre	0.085	0.014	0.212	0.012	14.554	1.713
2 post	0.061	0.016	0.206	0.018	14.319	1.751
3 pre	0.078	0.022	0.191	0.026	15.980	2.682
3 post	0.096	0.015	0.219	0.026	13.296	1.291

## Data Availability

All relevant data was included in the manuscript or added in the supporting information.
