# Supplementary material for: Objective Assessment of Porcine Voice Acoustics for Laryngeal Surgical Modeling
Source: Appl Sci (Basel). Author manuscript; Available in PMC 2022 Apr 28. (PMC9047298)
Supplement: Supplementary material [file NIHMS1794594-supplement-Supplementary_material.zip › applsci-1185445-supplementary/SupportingInformation/TableS2.pdf]

Table S2. Parameter abbreviations, expected change from pre- to post-surgery and reasoning behind this hypothesis.

Parameter	H1	Reasoning
PF	decreases	The share of high frequencies decreases after surgery. Therefore we also expect a decrease of PF as it becomes more likely that the most dominant frequency is lower.
Q50	decreases	Similar to PF, we also expect Q-Parameters to decrease due to a smaller share of high frequencies in the spectrum. The energy in the high frequencies decreases, resulting in a lower percentage of the total energy being in the high frequencies, which results in the 50% or 25% of the area being reached sooner.
$Q50_2$	decreases	
$Q50_{10}$	decreases	
$Q50_{min}$	decreases	
Q50W	decreases	
Q25	decreases	
Dur	decreases	After surgery the larynx is still damaged and requires time to heal. This may make phonation more strenuous and therefore result in shorter squeals.
$Q50_n$	decreases	We noticed that higher frequencies tend to vanish especially in the end of the signal in post-surgery pigs. Therefore, later partial widows should have a lower share in high frequencies. As $Q50_n$ denotes the partial window with the highest Q50 value we expect $Q50_n$ to decrease.
SF	increases	The decrease in energy in higher frequencies may result in a "flatter" spectrum in this frequency range. Therefore we expect Spectral Flatness to increase.
SF_{Q50}	increases	
Flux	increases	Squeals may become more noisy and chaotic after surgery, therefore we expect the change in energy between signal windows and therefore Flux to increase.
RMSI	increases	As the signal has less structure after surgery the relative sound intensity does change less over the course of the signal. This leads to a larger portion of the signal being closer to maximum height and hence may lead to an increased RMSI.
HNR	decreases	We expect squeals to become even less periodic after surgery and therefore HNR to decrease.
